# Medication Use During Breastfeeding—An Understudied Population: A Five‐Year Experience in the Pharmacovigilance Center of Lyon

**DOI:** 10.1002/cpt.70518

**Published:** 2026-10-06

**Authors:** Blandine Bertin, Aurore Gouraud, Anthony Facile, Fanny Ledys, Sabrina Pierre, Véronique Pizzoglio, Thierry Vial, Marine Auffret, Marina Atzenhoffer

**Affiliations:** ^1^ Pharmacovigilance Center, Hospital University Pharmacotoxicology Department Hospices civils de Lyon Lyon France

## Abstract

Medication use during breastfeeding is common, yet data on drug transfer into breast milk and infant exposure remain limited. This study describes real‐world requests concerning medication compatibility with breastfeeding managed by the Lyon pharmacovigilance center over five years. We conducted a descriptive observational study including all requests recorded between January 2019 and December 2023. Characteristics of infants, medications involved, and recommendations provided were analyzed. A total of 2,473 requests were included, corresponding to approximately 500 per year. Most requests originated from patients (44%), allied health professionals (29%), or specialists (25%), with two thirds occurring postpartum. Overall, our recommendations supported breastfeeding in 87% of cases, either unconditionally or under specific conditions, such as clinical monitoring, therapeutic adjustments, or timing strategies. Only 13% of cases resulted in an unfavorable recommendation, for either treatment or breastfeeding discontinuation, due to formal contraindications, insufficient therapeutic benefit, or limited safety data. Polytherapy was observed in 35% of cases and was associated with more restrictive recommendations. Medications used for chronic conditions accounted for 36% of cases such as antihypertensives, antiepileptics, or psychotropic agents. Treatments for acute conditions included anti‐infectives, analgesics, contrast agents, or anesthetics. Reported adverse effects in infants were rare and non‐serious. In most cases, a recommendation supporting breastfeeding during maternal treatment, with or without conditions, was provided. However, uncertainty persists, particularly in complex or poorly documented situations. This work highlights the need for reliable, accessible information, inclusion of breastfeeding women in clinical research, and strengthened regulatory and postmarketing evaluation strategies to ensure infant safety health.

**Study Highlights**

**WHAT IS THE CURRENT KNOWLEDGE ON THE TOPIC?**

Breastfeeding provides substantial benefits for both mothers and infants, yet despite the frequent use of medications during the postpartum period, data on drug transfer into breast milk and the safety of breastfed infants remain limited.

**WHAT QUESTION DID THIS STUDY ADDRESS?**

What are the characteristics of medication‐related breastfeeding requests received by a regional pharmacovigilance center, and what proportions of favorable, conditionally favorable, and unfavorable recommendations are provided in real‐world clinical practice?

**WHAT DOES THIS STUDY ADD TO OUR KNOWLEDGE?**

87% of Lyon PVC requests over 5 years result in recommendations supporting breastfeeding, either unconditionally or under specific conditions, emphasizing that most medications are considered compatible with lactation when appropriate precautions are taken.

**HOW MIGHT THIS CHANGE CLINICAL PHARMACOLOGY OR TRANSLATIONAL SCIENCE?**

These findings highlight the value of multidisciplinary expert consultation in translating pharmacological evidence into individualized, consensus‐based recommendations that support breastfeeding while ensuring maternal and infant safety.

Breastfeeding is a physiological process that provides substantial health benefits for both the infant and the mother. For the child, it is associated with a reduction in the risk of acute otitis media, non‐specific gastroenteritis, severe lower respiratory tract infections, atopic dermatitis, asthma (young children), obesity, type 1 and 2 diabetes, childhood leukemia, sudden infant death syndrome (SIDS), and necrotizing enterocolitis.^1^, ^2^ For the mother, it contributes to decreased risks of certain cancers and metabolic diseases.^3^ Consequently, international guidelines promote exclusive breastfeeding during the first six months of life, followed by the introduction of adequate complementary foods while continuing breastfeeding up to two years of age or thereafter according to individual circumstances.^2^, ^4^

Despite its benefits, breastfeeding remains an understudied area in clinical research, particularly with regard to medication safety. Although recent guidelines aimed at promoting the inclusion of pregnant and breastfeeding women in clinical trials,^5^ these populations are still frequently excluded from studies. Consequently, evidence regarding the safety of medications during breastfeeding remains scarce, with information on drug transfer into breast milk and its potential effects on breastfed infants often being limited or based on low‐quality evidence.

However, the use of medications during the breastfeeding period is common.^6^, ^7^, ^8^ Indeed, considering that approximately one in six pregnant women receives chronic medication and the 77% breastfeeding initiation rate reported in France, the number of women who breastfeed while receiving chronic medications is likely to be substantial.^9^, ^10^ Furthermore, others need to initiate therapy during breastfeeding for newly diagnosed conditions.^8^ In clinical practice, healthcare professionals frequently face uncertainty when advising breastfeeding women on medication use, which may lead to inconsistent recommendations, potentially leading to suboptimal maternal care, unnecessary treatment discontinuation, or premature cessation of breastfeeding.

Nevertheless, for the majority of medications, exposure through breast milk is low and compatible with breastfeeding when appropriate precautions are taken.^11^ Therefore, access to reliable information is essential to support informed decision‐making.

Pharmacovigilance centers (PVC) play a key role in providing individualized assessments of medication safety profile and compatibility during breastfeeding. Access to expert advice is therefore crucial to support individualized risk–benefit assessment and optimize both maternal treatment and breastfeeding continuation. These evaluations integrate multiple factors, including: (i) pharmacological characteristics, such as pharmacokinetic parameters (e.g., molecular weight, plasma half‐life, and oral bioavailability) and physicochemical properties (e.g., protein binding and lipid solubility); (ii) treatment‐related factors, including duration, dosage, and potential drug combinations; (iii) maternal clinical characteristics, including the underlying condition, available therapeutic alternatives, relevant medical history, exposure to other substances, and the benefit–risk balance of treatment for the mother; (iv) infant clinical characteristics, such as age, prematurity, birth weight, underlying medical conditions, and in utero drug exposure; (v) breastfeeding patterns, including frequency of feeds and exclusive or mixed breastfeeding; and (vi) social context, particularly the ability to monitor the infant and detect clinical signs.

In some cases, specific recommendations may be required including clinical or biological monitoring of the infant, therapeutic adjustments, measurement of drug concentrations in children's plasma or the mother's breast milk, or, in rare cases, temporary or permanent discontinuation of breastfeeding.

The Lyon PVC is nationally recognized for its expertise in this field as a member of the ENTIS network (European Network of Teratology Information Services) and is regularly consulted for such inquiries. Senior practitioners respond to requests received by email or telephone by providing individualized assessment of the medication's safety profile and compatibility with breastfeeding. All newly recruited practitioners receive dedicated training in lactation pharmacology, covering key factors involved in assessing medication compatibility with breastfeeding, and the conduct of a comprehensive literature search using specialized reference sources (e.g., Hale's Book,^12^ Sainte‐Justine University Hospital Editions^13^), bibliographic databases (e.g., LactMed,^14^ PubMed), and product information, and available pharmacokinetic data. In cases of uncertainty, a multidisciplinary discussion organized with other senior practitioners is convened to reach a collegial consensus. When therapeutic drug monitoring or clinical follow‐up of the breastfed infant is considered necessary, the requester is referred to an appropriate healthcare professional or service, such as a clinical laboratory, midwife, pediatrician, or maternal and child health center.

Describing real‐world requests addressed to our pharmacovigilance center may improve the understanding of medication exposure during breastfeeding, identify the most common clinical situations, and help prioritize future research in this field.

The aim of the present study was to describe the activity of the Lyon PVC in responding to requests concerning the compatibility of medications with breastfeeding over a five‐year period. Specifically, we analyzed the characteristics of the requests, the types of medications involved, and the recommendations provided, including the need for monitoring or therapeutic adjustments.

## MATERIAL AND METHODS

### Study design

This is a descriptive, observational, monocentric study based on data extracted from the local pharmacovigilance database, which records all requests received by the Lyon PVC. Requests concerning appropriate medication use during breastfeeding, as well as the responses provided by experts from our center, are recorded in this database.

Recorded variables for both mothers and infants (if born), in this database, were:

- Mothers' characteristics: medical condition, drug exposures during pregnancy and breastfeeding (indication, dosage, and treatment duration).
- Infants' characteristics: age or date of birth, prematurity (defined as gestational age <37 weeks), birth weight, medical condition.
- Breastfeeding condition: exclusive or mixed breastfeeding, number of feedings per day.

### Cases selection

We included all incoming requests related to breastfeeding received between January 1, 2019, and December 31, 2023.

Exclusion criteria were:

- Requests received during breastfeeding regarding an issue unrelated to breastfeeding.
- Insufficient data.
- Duplicate record.
- Cases outside the scope of the Lyon PVC expertise.
- Medication taken by the breastfeed infant.
- General question.

### Handling of data

Based on the information recorded in our database, we selected the following variables of interest:

- Type of inquiry (i.e., patient, healthcare professional, allied health professional).
- Timing of the request (during pregnancy, at delivery [from birth to 3 days postpartum], or during breastfeeding).
- Infant information (age, prematurity).
- PVC's advice regarding breastfeeding (favorable or unfavorable) and any PVC's recommendations (clinical and biological monitoring, drug dosing, breastfeeding interruption, mixed feeding).
- Medications involved, medication classifications, and treatment regimen (chronic or non‐chronic).

Recommendations were classified into three main categories, with additional subcategories: (i) favorable opinion for breastfeeding; (ii) breastfeeding permissible under specific conditions (clinical monitoring, treatment adjustment by using the lowest effective maternal dose or delaying the timing of the medication or proposed alternative, therapeutic drug monitoring in children, other ancillary tests in children, mixed feeding); (iii) breastfeeding not recommended with the use of the medication (formal contraindication, no benefit of treatment, lack of data and/or unfavorable safety profile, illicit drug use, polytherapy, no more benefit of breastfeeding, pediatric condition, social context, breast milk donation, adverse effects in the infant, maternal condition).

The drugs were grouped into 59 categories, which we classified by major drug class. A single call could involve multiple medications, hence the higher number of medications than requests.

Characteristics are described using frequency and percentage for categorical variables, and by mean (standard deviation) or median (interquartile range) for quantitative variables, as appropriate according to their distribution. The numbers of positive and negative responses were calculated for all medications combined and subsequently analyzed by individual drug class.

## RESULTS

### Study population and overall activity

The study was conducted over a 5‐year period (2019–2023). A total of 2,702 requests were extracted from the database. Out of these 2,705 requests, 2,473 were retained after excluding 232 based on the aforementioned exclusion criteria.

This corresponded to a mean of 495 requests per year (standard deviation [SD] 60).

### Type of inquiry

Most requests originated directly from patients (44%). Allied healthcare professionals including midwives and paramedical staff represented a substantial proportion of callers (29%), as well as specialists such as pediatricians, neonatologists, and psychiatrists (25%).

### Timing of requests

Regarding timing of requests, 66% of calls were made after childbirth, whereas 16% were received during pregnancy and 17% occurred around the time of delivery. Additionally, 1% of requests concerned questions related to breast milk donation. For postnatal calls, the median age of the infant was 3 months (interquartile range [IQR] 0.8–8; age available for *n* = 1,496).

Notably, 36% of requests concerned chronic treatments (information available for 2,469 cases).

Among requests received during pregnancy, 95% concerned chronic treatments, compared with 59% of chronic treatments for calls received at delivery and 16% of chronic treatments of those received during breastfeeding.

### Recommendation

Among the 2,473 requests evaluated, 847 (34%) were immediately supportive of breastfeeding, 1,310 (53%) provided guidance to enable it under defined conditions, and 316 (13%) recommended against the use of the medication during breastfeeding.

The recommendations included clinical monitoring (*n* = 847), therapeutic adjustments (e.g., dose reduction, timing adjustments between drug intake and breastfeeding, or alternative treatments) (*n* = 649), transition to mixed breastfeeding (*n* = 21), infant biological testing (*n* = 80), and drug concentration measurements in infant plasma and/or breast milk (*n* = 165).

These recommendations are presented in **Table** **1**.

**Table 1:** Recommendations to support breastfeeding under specific conditions

Recommendations	*n* = 1,310 (%)
Clinical monitoring	847 (65%)
Treatment adjustment or proposed alternative	649 (50%)
Therapeutic drug monitoring in children	165 (13%)
Other ancillary tests in children	80 (6%)
Mixed feeding	21 (2)

**Table** **2** illustrates the reasons for unfavorable recommendations to breastfeeding while taking the medication. The primary reason was a formal contraindication (28%) due to medications potentially harmful to the breastfed infants (e.g., opioid derivatives, cytotoxic agents). The other two major categories, each accounting for 25% of cases, were: the lack of therapeutic benefit leading to a recommendation to discontinue treatment (e.g., essential oils, dietary supplements, over‐the‐counter cold medications), and the lack of data for the use of a medication during breastfeeding and/or an unfavorable safety profile.

**Table 2:** Reasons for unfavorable recommendations to breastfeeding while taking the medication due to an unfavorable risk–benefit balance

Reasons for recommendations against breastfeeding while taking the medication	*n* = 316 (%)
Formal contraindication	88 (28%)
No benefit of treatment	80 (25%)
Lack of data and/or unfavorable safety profile	79 (25%)
Illicit drug use	17 (5%)
Polytherapy	15 (5%)
No more benefit of breastfeeding	12 (4%)
Pediatric condition	9 (3%)
Social context	7 (2%)
Breast milk donation	4 (1%)
Adverse effects in the infant	3 (1%)
Maternal condition	2 (1%)

### Number of medications

Of all 2,473 the requests, 65% concerned a single medication, while multiple‐medication regimens were documented in 35% of cases (two medications in 18% of cases, three in 9% of cases, and >3 medications in 8% of cases). **Figure** **1** shows the distribution of breastfeeding recommendation categories (favorable, conditionally favorable, or unfavorable) according to the number of medications involved. Favorable opinions were predominantly provided in situations involving a single medication, whereas conditional favorable opinions were more frequent in cases of multiple‐medication exposure. Although the proportion of unfavorable opinions remained relatively stable across groups, it increased from 12% for single‐medication exposures to 19% when more than three medications were involved.

**Figure 1:**
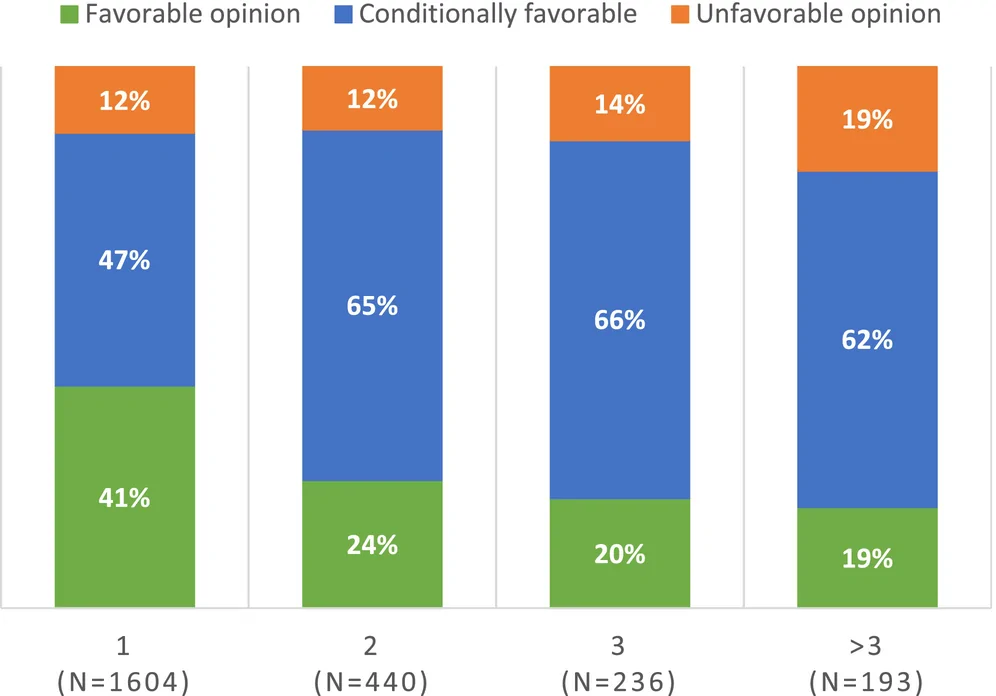
Distribution of breastfeeding recommendation category (favorable, conditionally favorable, or unfavorable) according to the number of medications involved.

### Preterm infants

A total of 340 requests concerned preterm infants. Among these, 101 cases (30%) received a favorable opinion and 92 (27%) received an unfavorable recommendation.

### Therapeutic classes

The most frequently involved therapeutic classes are presented in **Figure** **2**, along with details on the type of response provided (favorable, conditionally favorable, or unfavorable).

**Figure 2:**
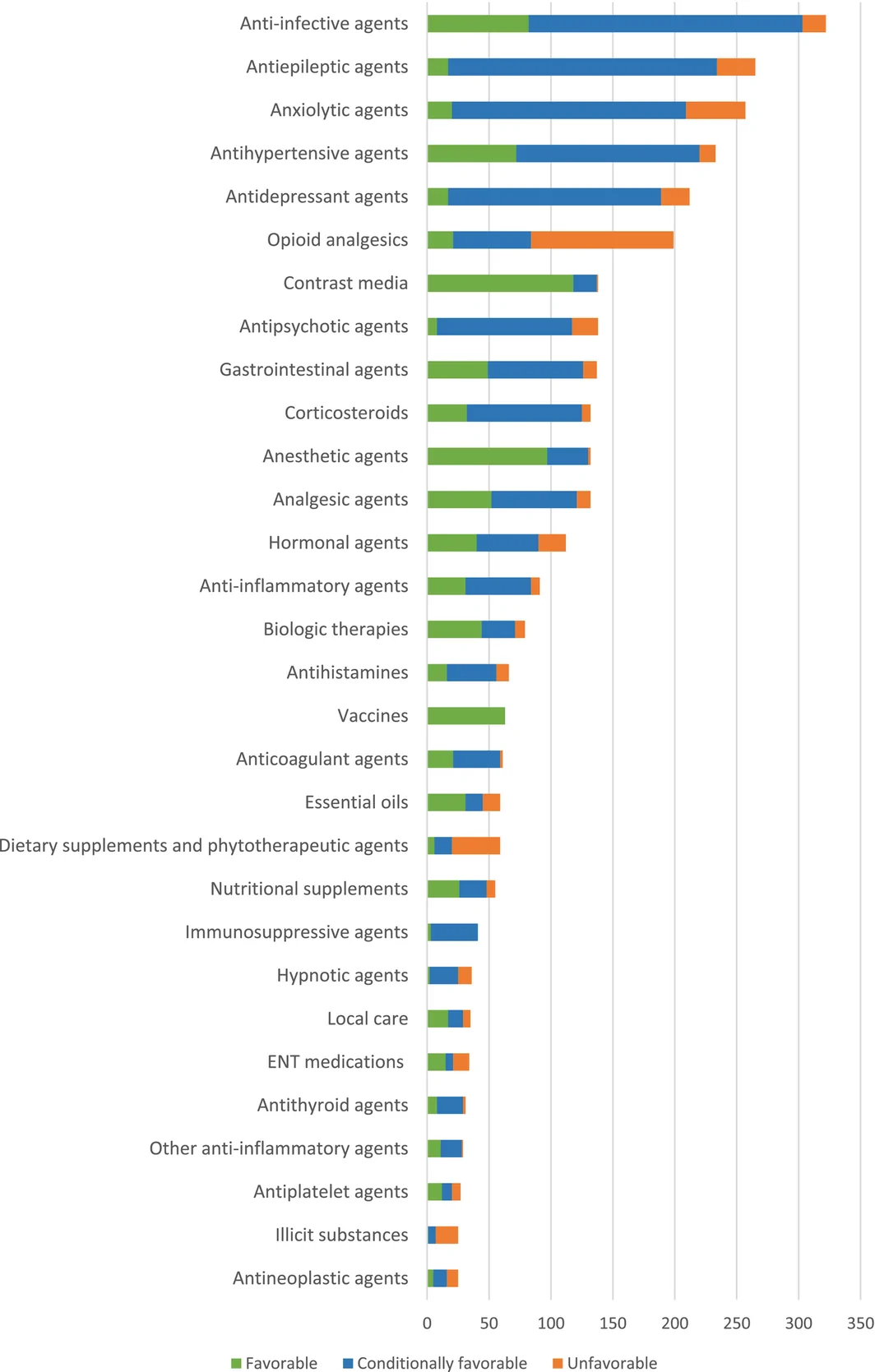
Distribution of therapeutic classes by response category.

No unfavorable recommendations were issued for immunosuppressive agents or vaccines. By contrast, unfavorable recommendations predominated for illicit substances (72%, *n* = 18/25), dietary supplements and phytotherapeutic agents (66%, *n* = 39/59), and opioid analgesics (58%, *n* = 115/199).

For illicit substances, favorable (*n* = 1) or conditionally (*n* = 6) recommendations were mainly associated with cases of drug‐facilitated assault or exposures that had been discontinued for a long time. Among opioids, favorable recommendations were largely limited to occasional or short‐term use, particularly tramadol. The high proportion of unfavorable recommendations for dietary supplements and phytotherapeutic agents was primarily driven by their insufficient therapeutic benefit. Antineoplastic agents were more often associated with conditionally favorable (44%) than unfavorable (36%) recommendations, with favorable opinions generally involving discontinued treatment or temporary interruption of breastfeeding. For hypnotics, 31% of recommendations were unfavorable, while 64% were conditionally favorable, mainly in situations where breastfeeding was avoided during the night; favorable recommendations generally concerned melatonin or occasional use.

All the therapeutic classes included in the analysis and their corresponding medications are listed in **Table** **S1**.

### Adverse events

Among over 100 followed cases (4% of the cohort), a total of seven adverse drug reactions (ADRs) were reported in infants and recorded in the French National Pharmacovigilance Database, representing 7% of the cases. The median age of the children was 2 months [IQR: 0.63–4]. All reported ADRs were non‐serious.

Three ADRs were reported with maternal vaccination: one case each of insomnia, fever with diarrhea, and regurgitation following administration of the COMIRNATY (Pfizer‐BioNTech) COVID‐19 vaccine. However, no formal causal relationship was established in these cases.

One case of abdominal pain was reported in a 15‐day‐old infant following maternal treatment with ofloxacin for pyelonephritis. The outcome remains unknown after switching to amoxicillin‐clavulanic acid.

One case of somnolence occurred in a 5‐day‐old infant following a 0.25 mg alprazolam intake by the mother, with rapid resolution.

A decrease in lactation was reported 8 days after initiation of desogestrel in a mother one month postpartum, without resolution after discontinuation of the contraceptive.

Finally, a case of moderate axial hypotonia was observed in a 3‐month‐old infant exposed to duloxetine through breastfeeding. The infant had been breastfed since birth and was described as poorly alert. Breastfeeding was discontinued, with subsequent improvement.

## DISCUSSION

This retrospective descriptive study, conducted over five years at the Lyon PVC, highlights the diverse demand for assessments of medication compatibility with breastfeeding. With 2,473 requests analyzed (approximately 500 per year), our findings confirm that, despite its physiological and health benefits, breastfeeding remains a major clinical challenge due to the lack of robust data on drug safety in this context.

Our data reveal that 44% of requests originated directly from mothers, reflecting an increasing trend toward patient autonomy in seeking reliable information. However, the majority of requests (54%) originated from allied healthcare professionals (29%) or specialists (25%), highlighting the persistent requirement for specialized guidance in complex clinical situations, such as breastfeeding while taking a new, rarely used medication or cases involving polypharmacy.

Another notable result concerns the timing of requests. Although our study population consisted of women seeking information about medication use during breastfeeding, only 16% of calls occurred during pregnancy. Among these prenatal requests, 95% concerned chronic treatments, highlighting an opportunity for anticipatory counseling and treatment optimization before delivery. Additionally, 17% of calls occurred at the time of delivery, often requiring urgent responses. Among these requests, 59% concerned chronic treatment, some of which might potentially have been anticipated during pregnancy.

These situations likely reflect a lack of prior planning from both patients and healthcare providers, combined with the stressful and emergent context of childbirth. This finding supports the need for better integration of medication counseling into routine prenatal care, particularly for women with pre‐existing medical conditions. Antenatal consultation could enable clinicians to anticipate potential issues, adjust treatments, and schedule clinical and laboratory monitoring for the newborn. Multidisciplinary discussions and expert assessments may optimize clinical management by balancing maternal treatment needs with infant safety. Regional Pharmacovigilance Centers can provide expert advice and facilitate coordination between healthcare professionals.

In contrast, the majority of requests (66%) were made after delivery, reflecting acute clinical situations or *de novo* treatment initiation (together accounting for 84% of cases) such as infections or postpartum depression, as well as the need to resume treatments for chronic conditions that had been discontinued during pregnancy. This highlights the reactive nature of many consultations and the need for immediate pharmacological intervention.

Polypharmacy was another key issue, present in 35% of cases, with 17% involving three or more medications. This complicates the assessment of drug safety during breastfeeding, as clinicians must consider not only individual drug properties but also potential drug–drug interactions, particularly pharmacodynamic interactions such as additive sedative effects. Furthermore, the extent of polypharmacy may be underestimated in our study, as not all medications are systematically coded in our local database.

Our findings (**Figure** **1**) suggest that polypharmacy may influence breastfeeding recommendations by shifting the balance from fully favorable opinions toward conditionally favorable ones. As the number of medications involved increased, unconditional approval of breastfeeding became less frequent and conditionally favorable recommendations more common. Although unfavorable opinions were often driven by a specific medication considered incompatible with breastfeeding, the proportion of unfavorable recommendations increased from 12% among women receiving up to three medications to 19% among those receiving more than three medications. This increase may suggest that extensive polypharmacy may also contribute to an unfavorable recommendation, particularly when several drugs with sedative properties or at high doses are involved. Overall, these findings suggest that polypharmacy primarily prompts a more cautious and individualized risk assessment rather than constituting a contraindication to breastfeeding.

Regarding the types of medications involved, both chronic and acute treatments were frequently represented. Chronic conditions were commonly managed with anxiolytic medications, antidepressants, antipsychotics, and antihypertensive drugs, while acute conditions often required antibiotics or contrast agents. These findings are consistent with the most prevalent health issues among breastfeeding women and highlight the importance of reliable data for frequently prescribed drug classes.

Reassuringly, when favorable and conditionally favorable recommendations were combined, breastfeeding was compatible with the medication in question in 87% of cases. This finding confirms that the majority of maternal treatments were considered to be compatible with breastfeeding by the PVC Lyon experts. However, in most cases (53%), specific precautions or recommendations were advised, such as clinical monitoring or dosage adjustments. These results support current evidence suggesting that breastfeeding should not be systematically discontinued when a mother requires pharmacological treatment.

In our study, 13% of recommendations advised were unfavorable to breastfeeding while taking the medication. Although this proportion may appear relatively high, it should be interpreted in light of the underlying reasons. As shown in **Table** **2**, a substantial proportion of these unfavorable recommendations was driven by situations in which no therapeutic benefit was expected, such as the use of essential oils, dietary supplements, or treatments for minor conditions. In these cases, discontinuation or avoidance of the medication was recommended, thereby preventing unnecessary exposure in the breastfed infant. Consequently, the decision to give an unfavorable opinion was not always related to intrinsic drug toxicity, but rather to a risk–benefit assessment in which the absence of maternal benefit outweighed any potential risk. This finding highlights the importance of critically evaluating the indication for treatment when considering medication use during breastfeeding.

Cases involving medications for which extensive data are available—such as anti‐infective, antihypertensive drugs, antidepressants, and anxiolytics—were predominantly associated with favorable assessments or considered compatible with breastfeeding provided that specific recommendations were followed.^15^, ^16^ Favorable responses were also most often provided for medications with reassuring safety profiles and no expected effects in the breastfed infant, including vaccines and contrast agents. Similarly, favorable opinions were issued for medications characterized by pharmacokinetic properties suggesting minimal infant exposure, such as monoclonal antibodies (classified as biological therapies in **Figure** **1**), topical agents with limited systemic absorption (or any local care), or anesthetics (many of which are characterized by relatively rapid elimination). Finally, for less well‐documented medications or drugs with non‐negligible transfer into breast milk, a clearly favorable benefit for the mother and limited risks for the infant may justify continuation of treatment (e.g., in the context of cystic fibrosis or certain antiepileptics, particularly lamotrigine), with a close clinical or biological monitoring of the infant.^17^, ^18^, ^19^

Conversely, high‐risk medications were contraindicated in the vast majority of cases. Opioids led to contraindications due to the risk of neonatal sedation and respiratory depression, and to the recommendation of temporarily interrupting breastfeeding, considering the drug's elimination half‐life to determine an appropriate washout period. Other less well‐characterized drugs with an unfavorable safety profile, such as cytotoxic or oral chemotherapy, were also associated with negative recommendations. For certain medications or substances, the relevance of their use during breastfeeding was questioned, including lipid‐lowering agents (given the importance of lipids for infant development), hypnotics (particularly in the context of nighttime breastfeeding).

For certain medications, the absence of data on their transfer into breast milk—or pharmacokinetic properties indicating potential significant excretion—may justify measuring drug concentrations in human milk. However, this approach faces substantial challenges, particularly the need to develop analytical matrices that simplify drug quantification in breast milk, a practice that remains uncommon today. In addition, plasma drug concentration measurements in the infant can provide valuable information on exposure, elimination capacity, and the risk of accumulation. In practice, this requires a blood sampling procedure in the infant and is therefore generally reserved for medications with substantial interindividual variability in milk transfer (e.g., lamotrigine) or for particularly sensitive or high‐risk situations (e.g., lithium).^20^, ^21^

Interestingly, our work shows that prematurity was not associated with a lower rate of favorable opinions compared with full‐term infants, suggesting that it should not be considered as an absolute contraindication to breastfeeding in the context of maternal medication use. However, within the group of unfavorable recommendations, a higher proportion was observed in preterm infants, with no apparent difference between chronic and acute/*de novo* treatments, likely reflecting a more cautious approach given their increased vulnerability to drug exposure.

The heterogeneity of the population further complicates interpretation. Differences in maternal underlying pathologies and social context mean that each case requires an individualized assessment. This variability limits the applicability of standardized recommendations and reinforces the need for tailored clinical decision‐making.

Several limitations should be acknowledged. First, missing data, particularly regarding the duration of breastfeeding in cases of chronic medication use, may limit the interpretation of long‐term exposure, although this factor did not directly influence the recommendations provided. Second, the absence of systematic or long‐term follow‐up precludes assessment of the actual impact of our recommendations on infant outcomes or breastfeeding continuation. Furthermore, adverse events may be underreported, as they are not systematically communicated back to our service.

Finally, variability in recommendations may be related to the ongoing evolution of knowledge regarding drug safety in breastfeeding.

Our findings are broadly consistent with the existing literature, which indicates that most medications are compatible with breastfeeding and that the benefits of breastfeeding generally outweigh potential risks when appropriate precautions are taken.^5^, ^6^ However, our study adds real‐world data on clinical practice in France, where such analyses remain limited.

These results have important implications for clinical practice. Improving healthcare professionals' knowledge about medication safety during breastfeeding, as well as increasing awareness of available risk assessment tools (such as LactMed database or Hale's book), could enhance patient counseling and reduce unnecessary treatment discontinuation or breastfeeding cessation.

While the development of standardized national protocols for common clinical scenarios (e.g., antidepressants or antibiotics) could reduce practice variability, such approaches must be balanced against the need for individualized care.

Future research should focus on prospective studies, including the integration of breastfeeding women into clinical trials, to better understand the impact of chronic or prolonged exposure to medications through breast milk. Additional studies quantifying drug transfer into breast milk and pharmacokinetic analyses in infants are also needed, particularly given the specific metabolic capacities of this population.

In conclusion, this study is, to our knowledge, the first in France to describe medication use during breastfeeding in a real‐world setting. The findings of the present study indicate that breastfeeding was considered compatible with maternal treatment in the vast majority of individual requests assessed by PVC Lyon experts. When necessary, tailored precautions were recommended to optimize the safety of both mother and breastfed infant. However, further research is essential to strengthen the knowledge and optimize clinical decision‐making in this field.

## FUNDING

No funding was received for this work.

## CONFLICT OF INTEREST

The authors declared no competing interests for this work.

## AUTHOR CONTRIBUTIONS

B.B. and M.A. wrote the manuscript; B.B., M.A., A.G., and M.A. designed the research; B.B., A.G., J.C., A.F., F.L., S.P., V.P., T.V., and M.A. performed the research; B.B. and M.A. analyzed the data.

## Supporting information


Table S1.
