# Supplementary material for: Medication Use During Breastfeeding—An Understudied Population: A Five‐Year Experience in the Pharmacovigilance Center of Lyon
Source: Clin Pharmacol Ther. 2026 Oct 6:10.1002/cpt.70518. Online ahead of print. doi: 10.1002/cpt.70518 (PMC13639764; doi:10.1002/cpt.70518)
Supplement: Supplementary file 1 [file CPT-9999-0-s001.docx]

**Supplementary Table S1. Index of Therapeutic Classes and Main Medications**

**Analgesic agents**

nefopam; nitrous oxide / oxygen; paracetamol; paracetamol / cafeine; tramadol

**Anesthetic agents**

articaine; atracurium; bupivacaine; curare; ketamine; lidocaine; lidocaine / prilocaine; lidocaine/adrenaline; mepivacaine; midazolam; oxybuprocaine; propofol; remifentanil; rocuronium; ropivacaine; sevoflurane; sufentanil; sugammadex

**Antiarrhythmic agents**

amiodarone; flecainide; mexiletine

**Antiasthmatic agents**

beclometasone; beclometasone / formoterol; budesonide / formoterol; fluticasone / vilanterol; ipratropium; montelukast; salbutamol; salmeterol / fluticasone; terbutaline; tiotropium; tiotropium / olodaterol

**Anticholinergic agents**

chlordiazepoxide / clidinium; fesoterodine; metixene; oxybutynine; scopolamine; trihexyphenidyl

**Anticoagulant agents**

antithrombine iii humaine; apixaban; calcium heparin; dalteparin; danaparoide; enoxaparin; fluindione; fondaparinux; heparin; nadraparine; rivaroxaban; tinzaparin; warfarin

**Antidepressant agents**

amitriptyline;citalopram; clomipramine; doxepin; duloxetine; escitalopram; fluoxetine; mianserin; mirtazapine; paroxetine; sertraline; venlafaxine; vortioxetine

**Antidiabetic agents**

insulin; metformine; repaglinide; sitagliptine

**Antiepileptic agents**

carbamazepine; eslicarbazepine; gabapentin; lacosamide; lamotrigine; levetiracetam; oxcarbazepine; perampanel; phenobarbital; pregabalin; sodium valproate; vigabatrin; zonisamide

**Antihistamines**

bilastine; cetirizine; desloratadine; dexchlorpheniramine; doxylamine; ebastine; fexofenadine; levocetirizine; rupatadine

**Antihypertensive agents**

acebutolol; amlodipine; atenolol; bisoprolol; candesartan; captopril; carvedilol; clonidine; enalapril; furosemide; hydrochlorothiazide; irbesartan / hydrochlorothiazide; labetalol; lercanidipine; losartan; methyldopa; metoprolol; nadolol; nebivolol; nicardipine; nifedipine; nimodipine; perindopril; propranolol; ramipril; sotalol; spironolactone; urapidil; valsartan; verapamil

**Anti-infective agents**

acyclovir; albendazole; amoxicillin; amoxicillin / clavulanic acid; amphotericine b; artemether / lumefantrine; azithromycin; benzyl benzoate; bictegravir; bictegravir / emtricitabine / tenofovir alafenamide; bifonazole; bismuth / metronidazole / tetracycline; caspofungine; cefazolin; cefixime; cefotaxime; cefpodoxime; ceftazidime; ceftriaxone; cefuroxime; ciclopirox; ciprofloxacin; clarithromycin; clindamycin; clotrimazole; cotrimoxazole; daptomycine; dexamethasone / ofloxacin; dihydroartemisinine / piperaquine; doxycycline; econazole; ertapenem; erythromycin; ethambutol; fidaxomicine; flubendazole; fluconazole; fosfomycine; fucidic acid; ganciclovir; gentamicin; isoniazide; ivermectine; josamycine; levofloxacin; linezolid; lymecycline; macrolide; metronidazole; miconazole; mupirocine; neomycine / polymyxine b / nystatine; nitrofurantoine; norfloxacin; nystatine; ofloxacin; penicillin; permethrin; piperacilline / tazobactam; pivmecillinam; praziquantel; pristinamycin; pyrimethamine; rifampicine; ritonavir; roxithromycin; secnidazole; sertaconazole; silver sulfadiazine; tedizolid; spiramycin; spiramycin / metronidazole; sulfamethoxazole / trimethoprim; temocillin; tenofovir; tenofovir disoproxil / emtricitabine; terbinafine; tigecycline; tinidazole; tobramycin; valaciclovir; vancomycin

**Anti-inflammatory agents**

acetylsalicylic acid (high dose); diclofenac; flufenamic acid; flurbiprofene; ibuprofene; ketoprofene; mefenamic acid; meloxicam; naproxene; niflumic acid; tiaprofenic acid

**Antimigraine agents**

eletriptan; oxetorone; sumatriptan; zolmitriptan

**Antineoplastic agents**

bleomycin; dacarbazine; doxorubicin; hydroxycarbamide; methotrexate; paclitaxel; tamoxifen; vinblastine; vincristine

**Antiplatelet agents**

acetylsalicylic acid (low dose); clopidogrel; ticagrelor

**Antipsychotic agents**

aripiprazole; chlorpromazine; cyamemazine; haloperidol; levomepromazine; lithium; loxapine; olanzapine; paliperidone; pipamperone; quetiapine; risperidone; zuclopenthixol

**Antithyroid agents**

benzylthiouracil; carbimazole; propylthiouracil; thiamazole

**Antivertigo agents**

acetylleucine; betahistine; meclizine

**Anxiolytic agents**

alprazolam; bromazepam; buspirone; clobazam; clonazepam; clorazepate; clotiazepam; diazepam; ethyle loflazepate; etifoxine; hydroxyzine; lorazepam; oxazepam; prazepam

**Biologic therapies**

adalimumab; aflibercept; anakinra; atezolizumab; bevacizumab; canakinumab; casirivimab / imdevimab; certolizumab; dupilumab; etanercept; golimumab; guselkumab; infliximab; mepolizumab; midazolam; natalizumab; nivolumab; ocrelizumab; ranibizumab; risankizumab; rituximab; sarilumab; secukinumab; sotrovimab; trastuzumab; ustekinumab; vedolizumab

**Bisphosphonates**

biphosphonate (not otherwise specified)

**Cholinergic agents**

neostigmine; pyridostigmine

**Contrast Medium**

ethyl esters of iodized fatty acids; gadobutrol; gadolinium; gadoteric acid; gadoteridol; iobitridol; iodixanol; iohexol; iopamidol; iopromide; ioversol; sulfur hexafluorure

**Corticosteroids**

betamethasone; budesonide; calcipotriol / betamethasone; clobetasol; cortisone; dexamethasone; fludrocortisone; fluticasone; hydrocortisone; methylprednisolone; mometasone; prednisolone; prednisone; triamcinolone

**Cosmetic products**

cosmetic (not otherwise specified)

**Cystic fibrosis therapies**

dornase alfa; elexacaftor / tezacaftor / ivacaftor; ivacaftor; tezacaftor / ivacaftor

**Dietary supplements and phytotherapeutic agents**

bromelains; cystine / methionine / vitamins; dietary supplement / phytotherapy (not otherwise specified); passion flower / valerian / hawthorn; selenium; spiruline; valerian; zinc

**Dopamine agonists**

bromocriptine; cabergoline; levodopa / benserazide; levodopa / carbidopa; pramipexole

**ENT medications**

alpha-amylase; amylmetacresol / dichlorobenzyl alcohol; brompheniramine; camphre / levomenthol / eucalyptus / thymol; carbocisteine; dextromethorphan; ent medications (not otherwise specified); helicidine; ibuprofene / pseudoephedrine; ivy leaf extract; lidocaine / enoxolone / erysimum; lysozyme / cetylpyridinium; oxomemazine; oxymetazoline; paracetamol / pheniramine / vitamin c; paracetamol / pseudoephedrine; tixocortol; tuaminoheptane / acetylcysteine

**Erythropoiesis-stimulating agents**

darbepoetine alfa

**Essential Oils**

camphre; camphre / menthol; essential oils (not otherwise specified)

**Gastrointestinal agents**

alverine / simethicone; benzocaine / esculosin / dodecyclonium / enoxolone; betaine; diosmectite; domperidone; droperidol; esomeprazole; fluocortolone / cinchocaine; lactulose; lansoprazole; loperamide; macrogol; macrogol / electrolytes; mesalazine; metoclopramide; metopimazine; omeprazole; ondansetron; pancreatine; pantoprazole; paraffine liquide; phloroglucinol; pinaverium; prednisolone / cinchocaine; rabeprazole; racecadotril; ranitidine; saccharomyces boulardii; sodium alginate / sodium bicarbonate; sodium picosulfate / magnesium citrate; sodium, magnesium, and potassium sulfates; sulfasalazine; trimebutine; ursodeoxycholic acid

**Hemostatic agents**

eltrombopag; romiplostim; tranexamic acid; von willebrand factor

**Homeopathic preparations**

homeopathy (not otherwise specified)

**Hormonal agents**

cetrorelix; chlormadinone; choriogonadotropine alfa; clomifene; danazol; desogestrel; dienogest; dydrogesterone; estradiol; follitropine alfa; follitropine alfa / lutropine alfa; follitropine beta; ganirelix; gemeprost; glucagon; lactobacillus / estriol / progesterone; leuprorelin; levonorgestrel; levonorgestrel / ethinylestradiol; levothyroxine; levothyroxine / liothyronine; menotropine; mifepristone; misoprostol; oestro-progestatif; oral contraceptive (not otherwise specified); oxytocine; progesterone; progestogen; promegestone; promestriene; sulprostone; triptorelin; ulipristal

**Hypnotic agents**

alimemazine; loprazolam; lormetazepam; melatonin; zolpidem; zopiclone

**Illicit substances**

cannabidiol; cannabis; cannabis medical; cocaine; hallucinogenic mushroom; np-cannabinoides naturels

**Immunosuppressive agents**

azathioprine; cyclosporine; mycophenolate mofetil; tacrolimus

**Iodine**

iode; iode 131

**Lipid-lowering agents**

atorvastatine; colestyramine; ezetimibe; ezetimibe / simvastatine; rosuvastatine; simvastatine

**Local care**

chlorhexidine; chlorhexidine / cetylpyridinium; chlorhexidine / chlorobutanol; chlorhexidine / hexamidine / chlorocresol; dexpanthenol; hexamidine; hexamidine / lidocaine; hydroquinone; imiquimod; levomenthol / veratrole / resorcinol; malathion / permethrin; methyle salicylate / magnesium sulfate; methylrosanilinium; minocycline; minoxidil; povidone iodee; retinoides; salicylic acid; salicylic acid / lactic acid

**Multiple sclerosis therapies**

dimethyl fumarate; diroximel fumarate; fingolimod; glatiramere acetate; interferon beta-1a; natalizumab; peginterferon beta-1a

**Muscle relaxants**

baclofen; botulinum toxin; methocarbamol; thiocolchicoside

**Nutritional supplements**

alfacalcidol; calcidiol; calcium / vitamin d3; calcium carbonate; calcium folinate; cystine b6; ferrous sulfate; ferrous sulfate / vitamin c; fluoride; folic acid; fumarate ferreux; iron; magnesium; magnesium / vitamin b6; multivitamins; nutritional supplements (not otherwise specified); potassium chloride; sodium phosphate; tocopherol; vitamin b1; vitamin b12; vitamin b6; vitamin c; vitamin d; vitamin d3

**Ophthalmic preparations**

apraclonidine; atropine; borax / boric acid; brinzolamide / timolol; carteol; cyclopentolate; dexamethasone; hexamidine; latanoprost; phenylephrine; rifampicin eye drops; spaglumic acid; tobramycin / dexamethasone; tropicamide

**Opioid agents**

buprenorphine; methadone

**Opioid analgesics**

codeine; fentanyl; morphine; nalbuphine; oxycodone; paracetamol / codeine; paracetamol / opium; paracetamol / opium / cafeine; paracetamol / tramadol; tramadol

**Organic nitrates**

isosorbide dinitrate; molsidomine; nicorandil

**Other anti-inflammatory agents**

colchicine; hydroxychloroquine

**Other cardiovascular agents**

heptaminol; naftidrofuryl; purified micronized flavonoid fraction

**Other imaging agents**

fluorescein; indocyanine green; technecium

**Others**

allergenic extract; botulinum toxin; calcium polystyrene sulfonate; glycerol; hyaluronic acid; icatibant; mercury; peginterferon alfa-2a; pentosan polysulfate; puva therapy; quinine / hawthorn; sodium bicarbonate; sodium polystyrene sulfonate; trientine

**Plasma-derived medicinal products**

albumin; human immunoglobulin

**Psychostimulant agents**

methylphenidate; modafinil; pitolisant

**Sclerosing agents**

lauromacrogol 400; varicose vein sclerosant

**Tyrosine kinase inhibitors**

baricitinib; imatinib; nintedanib

**Vaccines**

covid-19 vaccine; influenza vaccine; mmr vaccine; varicella vaccine
